# A New Decentralized Robust Secondary Control for Smart Islanded Microgrids

**DOI:** 10.3390/s22228709

**Published:** 2022-11-11

**Authors:** Ali M. Jasim, Basil H. Jasim, Vladimír Bureš, Peter Mikulecký

**Affiliations:** 1 Electrical Engineering Department, University of Basrah, Basrah 61001, Iraq; 2 Department of Communications Engineering, Iraq University College, Basrah 61001, Iraq; 3 Department of Information Technologies, Faculty of Informatics and Management, University of Hradec Králové, 500 03 Hradec Králové, Czech Republic

**Keywords:** microgrid, distribution generators, secondary control, genetic algorithm, artificial neural network, virtual impedance, power sharing

## Abstract

Dealing with the islanded operation of a microgrid (MG), the micro sources must cooperate autonomously to regulate the voltage and frequency of the local power grid. Droop controller-based primary control is a method typically used to self-regulate voltage and frequency. The first problem of the droop method is that in a steady state, the microgrid’s frequency and voltage deviate from their nominal values. The second concerns the power-sharing issue related to mismatched power line impedances between Distribution Generators (DGs) and MGs. A Secondary Control Unit (SCU) must be used as a high-level controller for droop-based primary control to address the first problem. This paper proposed a decentralized SCU scheme to deal with this issue using optimized PI controllers based on a Genetic Algorithm (GA) and Artificial Neural Networks (ANNs). The GA provides the appropriate adjustment parameters for all adopted PI controllers in the primary control-based voltage and current control loops and SCU-based voltage and frequency loops. ANNs are additionally activated in SCUs to provide precise online control parameter modification. In the proposed control structure, a virtual impedance method is adopted in the primary control scheme to address the power-sharing problem of parallel DGs. Further, in this paper, one of the main objectives includes electricity transmission over long distances using Low-Voltage DC Transmission (LVDCT) systems to reduce power losses and eradicate reactive power problems. Voltage Source Inverters (VSIs) are adopted to convert the DC electrical energy into AC near the consumer loads. The simulation results illustrated the feasibility of the proposed solutions in restoring voltage and frequency deviations, reducing line losses, as well as achieving active and reactive power sharing among the DGs connected to the MG.

## 1. Introduction

Recent advances in power system technology have spurred significant transitions toward sustainable, modern, and intelligent grids. As newly emerging small-scale grids, MGs can accommodate a variety of technologies, such as Renewable Energy Resources (RERs), energy storage, power electronic devices, and demand management programs. Since most RERs are intrinsically DC or DC-friendly, integrating these sources with a long DC Transmission (DCT) is simple. Due to their high efficiency, high reliability, and simple integration with renewable energy sources, Low-Voltage DCT (LVDCT) systems appear to be an attractive option for distribution systems. In the MG system, to reduce power losses and eliminate reactive power issues, long-distance LVDCT transmission of electricity is preferred for supplying VSIs. In contrast, short-distance AC transmission lines can be used to supply three-phase MG loads. MGs have many benefits, such as using more than one type of DG, using renewable energy, reducing pollution, and increasing economic benefits [1,2,3,4]. Due to their decentralized nature, MGs can be installed in remote locations and improve network reliability.

**Figure 1:**
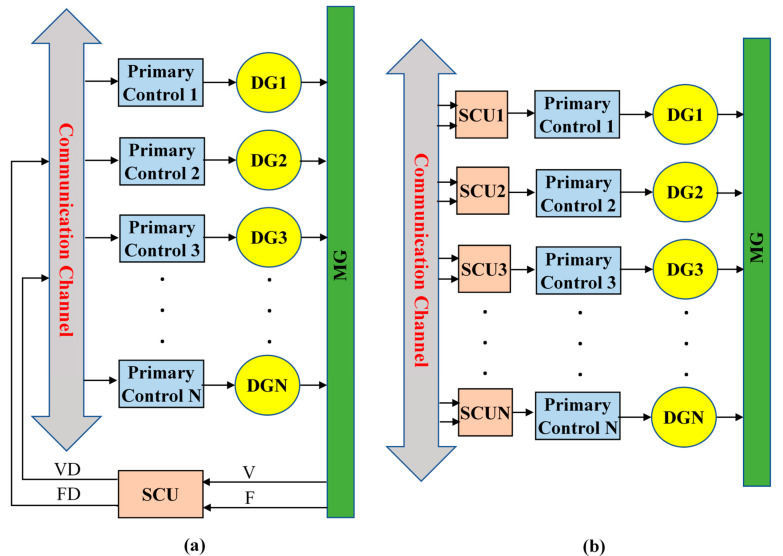
(**a**) Centralized and (**b**) decentralized secondary control strategies.

The term “smart MGs” refers to electrical networks that use digital and other modern technology to monitor and control the conveyance of power from all generating sources to satisfy shifting end-user electricity needs. In a smart MG, the resources and capacities of the various power plants, grid managers, and consumers are coordinated. Artificial Intelligence (AI) has been used in several smart MG-related disciplines, such as control approaches, security and reliability evaluation, energy management systems, data-driven decision-making systems, and so on. AI is a prominent field of computer science. AI can make optimal use of existing data and aid in making judgments in difficult situations for safer and more reliable MG control and operation. AI includes machine learning (ML). ML models are supervised or unsupervised based on training data. In MGs, system control and monitoring need an enhanced methodology that blends data-driven modelling to solve observability and controllability challenges [5]. MG hierarchical control schemes include numerous control levels based on functionality [6]. AI approaches may improve MG control and operation accuracy, speed, and effectiveness [7]. The MG can operate in off-grid “island mode” in addition to the grid-connected mode. The Q-V and P-f droop control methods are commonly used to achieve power sharing among all DGs. Active and reactive power sharing is gained when P-f and Q-V control are used, but the frequency and voltage values in the steady state are not always at nominal ones. As a result, an intelligent SCU is required to adjust the grid’s voltage and frequency [8,9]. SCUs can also be used to compensate for reactive power [10]. In general, SCUs may be decentralized or centralized, as depicted in Figure 1. In a centralized control strategy, the voltage and frequency of the grid are typically estimated and compared to their respective reference values. The error signals are processed by a controller, which then transmits correcting signals to all DG units (see Figure 1a). Figure 1b depicts a typical decentralized control; in this case, every DG unit is outfitted with SCU to correct voltage and frequency deviations.

The general configuration of an islanded MG consists of several DGs, each of which is connected to the load via a Power Electronic (PE) interface. This interfacing is typically accomplished with a nonlinear PE device, such as a PWM-based converter adopted to connect DGs within MG [11]. The primary issue with these devices is that they generate nonlinearity between current and voltage, producing high switching frequency pulses that distort the power quality [12]. As a result, MG faces significant power quality challenges, especially when incorporating an excessive number of DGs [13,14]. A reliable method of control is typically necessary to meet power quality standards and keep the MG system running smoothly. Large power, frequency, and voltage variations occur in the islanded mode of MG operation compared to the grid-connected mode due to the lack of inertia and uncertainty in selecting optimal gains of the Proportional Integral (PI) controller. Due to these issues, this research is being conducted to enhance MG’s functionality in the islanded mode of operation.

Nevertheless, a significant shortcoming of PI controllers is their limited performance, which relies heavily on fine-tuning the proportional and integral gain coefficients (K_p_ and K_i_) [15]. Throughout the process, these coefficients can be made static or dynamic using soft-computing techniques. Adaptive or “trial and error” methods [16,17,18], or the alternative Ziegler–Nichols (Z–N) method [19,20,21], are used to calculate static gains in PI regulators used for control loops that employ static gains. Because of this, they may delay the transition into a stable operating region [22]. Correct tuning of PI gains is crucial and challenging in order to guarantee improved system performance and power quality during DG incorporation and load changes [23].

ANN’s training and expandable characteristics enable the system to manage changes and uncertainty. Learning algorithms overcome technical obstacles. Online tuning controllers improve secondary voltage output and frequency regulation. Early secondary MG controller research resulted in classical PI controllers [24,25,26]. The main reasons for these controllers’ industrial success are their simplicity and ease of implementation. However, they are less dependable and robust due to their reliance on operating point conditions. The technical bottlenecks can be overcome with the help of intelligent learning algorithms. Tuning controllers that are both online and robust has a significant impact on the control of secondary frequency and voltage.

In the literature, different ways to choose the controllers’ coefficients have been outlined [27]. Most of them rely on trial-and-error methods, which can be time-consuming when dealing with a complex MG and often result in less-than-optimal tuned parameters. Further, this method does not provide a systematic way to design the coefficients of the controllers in the MGs. Another way is to set the parameters of the controllers so that the outer loop is slower than the inner loop [28]. Based on this idea, both the inner and the outer control loops can be constructed in a standalone fashion. Most of the time, the outer loop’s bandwidth is only 0.1 of the inner loop’s [29]. Further, this method has the same problems as the trial-and-error method. For the purpose of restoring the average voltage without the need for extra communication links, the authors of [30] suggested a secondary voltage control technique that makes use of state estimation in autonomous MG neighboring. In reference [31], a distributed-averaging-proportion-integral (DAPI) controller is proposed to address the adjustable tradeoff between voltage restoration and accurate reactive power sharing. The majority of the existing literature on distributed secondary control offers solutions for active power sharing and frequency/voltage recovery with asymptotic convergence speeds. Secondary control based on PI controllers and consensus observers for reactive power sharing and voltage restoration is proposed in [32]. The PI controller parameters are chosen by trial and error and no intelligent or optimization algorithms are adopted. Furthermore, if the optimal control settings are used and the islanded MG may be stable through several operations with less frequency and voltage steady-state errors. Parameters of the PI controller were tuned using a Grasshopper Optimization Algorithm (GOA) in [33] and PSO in [34]. The parameters of a triple-action controller for an AC islanded MG were designed using PSO in [35]. To regulate an isolated MG’s supply voltage and frequency, the authors of reference [36] developed a PSO-based controller. The developed controller optimized the system’s dynamic response with respect to regulating voltage within the prescribed limit (5% of the rated value), but the frequency response exceeded the limit (1% of the rated value) before stabilizing. The PSO-based controller in [37] was developed specifically for island MG. In spite of significant variations in both source and load, the controller maintained a frequency well within the allowed range. Based on previously published research papers, the authors of [38] assessed robust control approaches for MGs, comprising DC, AC, and hybrid MGs, with various topologies and forms of connectivity to conventional power systems. To regulate the AC voltage in AC MGs, [39] created a robust controller using the Cohen-Coon (CC) tuning technique. In [40], a PID robust secondary controller frequency control for an isolated AC MG is presented as a means of dealing with the inherent unpredictability of the energy supply from renewable energy sources. In order to restore the AC voltage and frequency in isolated AC MGs, [41] created a completely distributed secondary control (DSC) approach. Multiple studies are undertaken to find the optimal power distribution for MGs. Power distribution in spatially concentrated AC MGs with grid connection were addressed in [42], which details the development of a hierarchical controller with two layers. The goal of [43] is to improve power sharing in electrical networks that include both traditional power sources and DERs by proposing an enhanced droop-based control method. In [44], a control approach is developed that considers battery protection and reactive-power variations in a network bus, relying on a battery supercapacitor power system for the benefit of both storage systems. These studies do not address the power-sharing issue in an islanded MG and the LVDCT system is not adopted.

This study focuses on islanded smart MGs and tries to improve the secondary control mechanism to reduce voltage and frequency deviations. An intelligent control strategy adjusts the control settings to retrieve these parameters at the nominal levels. MG’s decentralized controller has an online self-optimizing control technique. In the tuning procedure, GA provides the first parameter adjustments. Then, an ANN modifies control settings online. ANN’s training and extensible features eliminate the controller’s reliance on operating point circumstances, allowing the system to handle changes and uncertainty. AC MG stability is of paramount importance due to the sensitivity of frequency and voltage deviations to the variations in MG loads. Moreover, different AC transmission line impedances lead to unequal power sharing between the parallel VSIs due to their inherent characteristics. To the authors’ knowledge, no prior research has discussed the use of decentralized SCUs utilizing GA-optimized ANN-based PI controllers to simultaneously restore frequency/voltage as well as equally share active/reactive power over LVDCT. The LVDCT reduces power losses and eliminates reactive power issues over long distances. To validate the proposed technique, a variety of case studies, including system load changes, without adopting SCU and using SCU, are simulated using MATLAB R2020a software in the islanded mode of operation. The paper’s contributions are listed below.

1. This paper proposes an effective control process with droop, inner, and SCUs for voltage and frequency control to improve MG performance. The proposed approach adjusts voltage and frequency in the MG assembly automatically in real time.
2. To handle the loading variations and improve power quality, we create a robust online fine-tuning strategy based on decentralized secondary control using ANN learning features. The parameter tuning for an SCU in an MG application can be tuned online using a combination of ANN and GA. The optimal secondary PI parameters are determined by GA and stored in the SCUs permanently. The modification of the PI controller-based GA parameters by an online ANN co-occurs at any time after the starting simulation. The proposed control mechanism’s extensibility is enhanced by the ANN controller’s ability to learn, creating an independent online controller.
3. The proposed control strategy makes use of the primary control-based virtual impedance method for satisfying the power-sharing requirements.
4. A long-distance LVDCT transmission system is adopted for powering VSIs since it reduces power losses and eliminates reactive power problems. Short-distance AC transmission lines are used to power three-phase MG loads.

This paper’s remaining sections are arranged as follows: The proposed MG system is elaborately discussed in Section 2. The modeling of system-generation resources is developed in Section 3. Section 4 contains the specifics of PI control parameter tuning. Section 5 describes the decentralized secondary control formulation. Section 6 conducts extensive simulation studies to evaluate the performance of the proposed controller. The paper is concluded in Section 7. 

## 2. Proposed MG System

**Figure 2:**
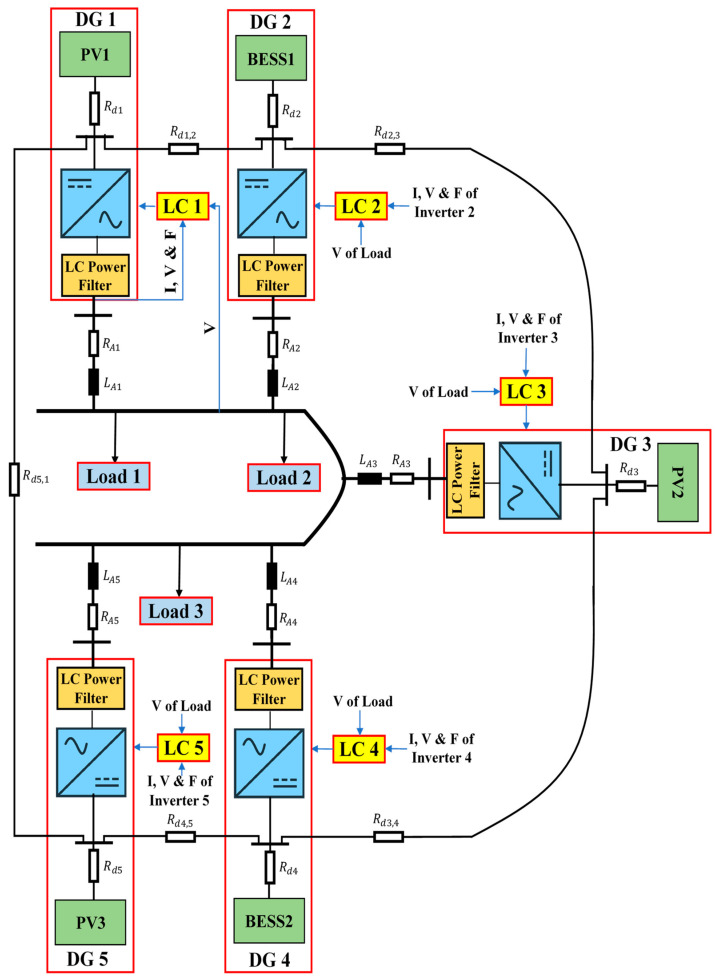
The adopted islanded MG.

**Figure 3:**
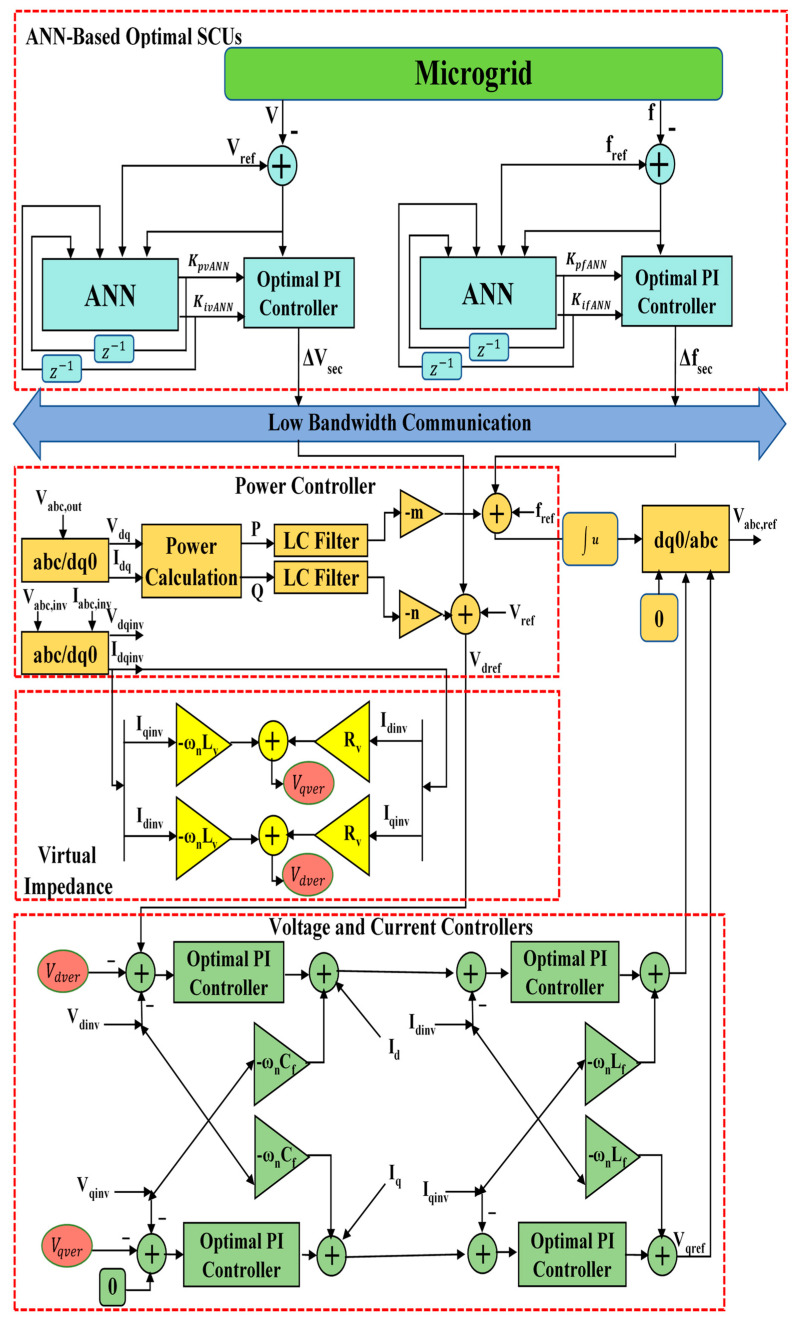
The control structure of each DG.

**Figure 4:**
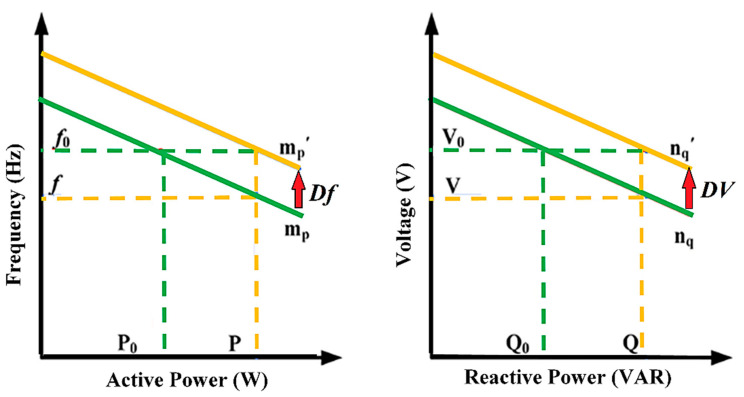
P/f and Q/V droop characteristics.

Consider the adopted islanded MG indicated in Figure 2. This MG is made up of five 700 V DC sources (three solar Photovoltaics (PVs) and two Battery Energy Storage Systems (BESSs)), five VSI-interfaced DGs (providing load power according to their capacity), ten DC line impedances, five AC transmission line impedances, shown in Table 1 and Table 2, showing droop coefficients and power filter parameters for each VSI, as well as three load banks shown in Table 3, with their time-value-based active and reactive load power. Active and reactive power losses are the primary issue with transmission lines. It is critical to resolve this issue or the majority of electrical energy will be lost in the transmission system. In this paper, low-voltage DC transmission lines are used to transfer electricity from MG’s DC sources into associated VSIs because they minimize power losses and eliminate reactive power issues. Some of the benefits of DC transmission include enhancing grid performance and protecting against cascading blackouts; environmental friendliness; distances are not limited by stability; and no need for reactive power compensation [45]. The direct resistances of the DC lines for connecting DC sources with related VSIs are represented by [ $R_{d1},R_{d2},\;R_{d3},R_{d4},\;R_{d5}$ ]. The between DC lines’ impedances [ $R_{d1,2},R_{d2,3},\;R_{d3,4},R_{d4,5},\;R_{d5,1}$ ] are inserted to make the grid more reliable in the event that a single DC power source has an outage or requires repair. Localized AC transmission lines drive three-phase MG loads with impedances denoted by [ $R_{A1}+L_{A1},R_{A2}+L_{A2},\;R_{A3}+L_{A3},R_{A4}+L_{A4},\;R_{A5}+L_{A5}$ ]. Figure 3 illustrates the control structure scheme of each DG. This control architecture comprises primary and secondary control layers. This structure’s measured signals are all in the d-q frame. The power part of each DG consists of an inverter, an output power filter, and the coupling inductor. The three parts of each local primary control scheme are power, voltage, and current controllers. The power control loop establishes reference points for the inverter’s output voltage and frequency based on the droop characteristics of P/f and Q/V. The frequency and voltage droop coefficients are $m_{p}$ and $n_{q}$. The droop controller concepts are presented in Figure 3 and Figure 4, which are (P/f, Q/V). The droop controller concepts are presented in Figure 4, which are (P/f, Q/V). The slopes of the droop controller characteristics have an effect on the system’s stability; thus, they need to be adjusted such that active and reactive power are shared equally. Large slopes may be employed to increase the rate of load sharing, but at the expense of the overall system’s stability. However, with droop control, the steady-state frequency and voltage values are not always at nominal levels; they drop at (V, f). Therefore, the droop controller has to be enhanced by applying a suitable secondary controller to compensate the frequency and voltage deviations (DV, Df) and restore the system voltage and frequency to their nominal levels (V_o_, f_o_) [46,47]. Power’s instantaneous active (P) and reactive (Q) components are injected through low-pass filters with cut-off frequencies of 10π to remove rapid fluctuations from power calculations. The virtual impedance method with (virtual resistor = 0.03 Ω and virtual inductor = 0.57 Ω) is adopted to mitigate the line impedance of each DG with the MG. The power controller and virtual impedance loops generate reference voltage and frequency, as well as line impedance’ voltage drops, which are fed to voltage and current controllers to generate reference voltages and currents of inverters in the dq0 reference frame. Both current and voltage controllers are intended to remove persistent disturbances while also dampening the output filter.

Because the droop controller cannot return the system frequency and voltage to their nominal values, it is necessary to use an appropriate controller, such as a secondary controller, to improve the droop controller method [48]. The secondary controller is responsible for correcting steady-state errors that the droop controllers have ignored. The implemented secondary GA and ANN-based frequency and voltage controllers are shown in Figure 3. The frequency signal is measured instantly and then evaluated by comparing to its reference value, as previously mentioned. A power controller is used to supplement the signal released by the appropriate PI controller. The method used to control the voltage is very similar. The PI controllers’ parameters are adjusted to make the output voltage as stable as possible. The secondary controller supervises the load end voltage and frequency and produces a signal to supplement the DGs’ control set-points. Notably, the reference points are only slightly modified by the control signals from the SCUs. Thus, they serve to supplement the primary control’s established limits. Therefore, accurate measurements are carried out in an attempt to lessen load voltage and frequency fluctuations. When it comes to ensuring the MG assembly runs smoothly and without incident, SCUs are a crucial control process.

## 3. System-Generation Resource Modeling

### 3.1. Solar Photovoltaic

The equation that defines the I–V behavior of the PV cell circuit model with one diode and two resistors is given in Equation (1) [49,50].

(1) $$I=I_{PV}-I_{O}\left\{\exp\left(\frac{V+IR_{s}}{\alpha V_{T}}\right)-1\right\}-\frac{V+IR_{s}}{R_{sh}}\;$$

where $I_{PV}$ is the photocurrent, $I_{O}$ is the reverse saturation current of the diode, $R_{s}$ is the series resistor that accounts for losses in cell solder bonds, junction box, interconnection, and other components, and $R_{sh}$ is the shunt resistor that takes into account current leakage through the highly conductive shunts across the p–n junction, whereas α is the ideality factor that describes how far the diodes deviate from their ideal state; $V_{T}$ is the thermal voltage of the diode and it is influenced by the number of series-connected cells (*n*), the electron charge (*q*), the Boltzmann constant (*k*), and the temperature (*T*) of the diode:

(2) $$V_{T}=n\frac{kT}{q}\;$$

### 3.2. Battery Energy Storage System

The terminal voltage and the SOC of a BESS are two crucial factors to represent the battery state and they are represented as follows [51]:

(3) $$V_{o}=V_{b}+R_{b}i_{b}-K\frac{Q}{Q+\int^{\;}i_{b}dt}+A.e^{B\int^{\;}i_{b}dt}\;$$

(4) $$SOC=100\left(1+\frac{\int^{\;}i_{b}dt}{Q}\right)$$

where $R_{b}$ denotes the internal battery resistance, $V_{o}$ represents the open circuit battery voltage, $i_{b}$ is the BESS charging current, K is the polarization voltage, Q is the capacity of the battery, A is the exponential voltage, and B is the capacity of the battery.

## 4. Tuning of PI Control Parameters

As stated, each DG needs two inner controllers for the voltage and current and frequency and voltage secondary feedback loops. The proportional gain and integral gain coefficients are observed in every controller. As a result, there are a total of eight control coefficients. The ability to adjust the parameters over a larger operating range is made possible by the use of such evolutionary algorithms as GAs. GAs are adaptable enough to work on multiple strings at once, each representing a unique solution to a particular problem. Consequently, the search space is meticulously scanned. Higher confidence levels are associated with the outcomes, drastically reducing the likelihood of hitting a local minimum [52].

**Figure 5:**
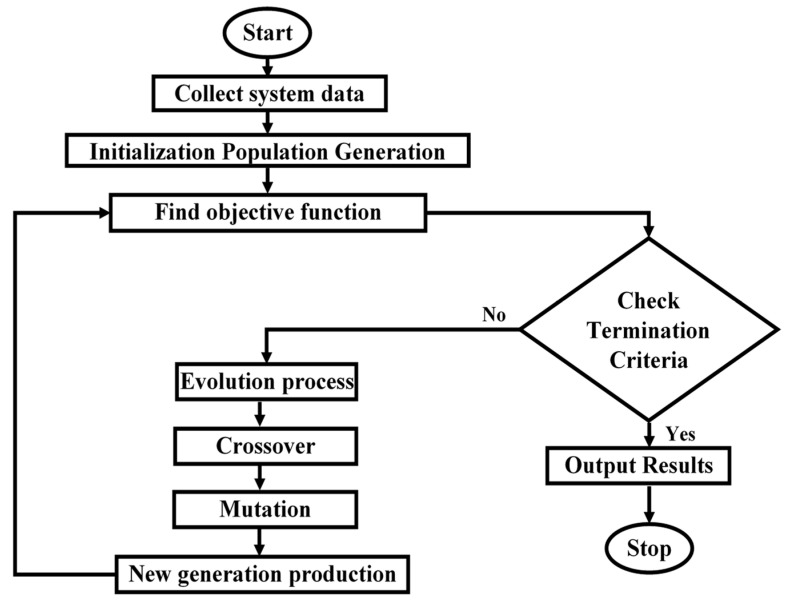
Flowchart of the GA-based optimization procedure.

We set all eight parameters for all DGs to the same value. Applying GA to the proposed system, the initial search space is constrained. On the basis of the ranges of control parameter ranges, we establish a suitable range to provide adequate liberty for the GA and a satisfying control procedure. In this model, a string serves as the chromosome containing eight genes coincident with their respective parameters. The following objective function (O.F) is used to restore the frequency and voltage signals to their target values:

(5) $$O.F=\sqrt{\begin{array}{c} \left\{\sum_{n=1}^{N}{\left(k_{vl}\left|\left(\left(\sqrt{{\left|v_{dLoad}\left(n\right)\right|}^{2}+{\left|v_{qLoad}\left(n\right)\right|}^{2}}\right)-v_{nomAC}\left(n\right)\right)\right|\right)}^{2}\right\}\;\\ +\left\{\sum_{n=1}^{N}k_{f}{\left|f_{nom}\left(n\right)-f\left(n\right)\right|}^{2}\right\}+\left\{\sum_{n=1}^{N}k_{vo}{\left|vo\left(n\right)\right|}^{2}\right\} \end{array}}$$

where N is the samples of the simulation time, n is the sample number, $v_{dLoad}$ and $v_{qLoad}$ are the dq components of the load voltage, $v_{nomAC}$ is the nominal AC voltage of the MG system, and $f_{nom}$ and f are the nominal and measured system frequency. The third term of the objective function (vo) is related to the inverter output voltage and it exists if and only if this voltage exceeded the nominal value. There are upper voltage and frequency deviation limits in a low-voltage, 50 Hz, 220 V distribution system, respectively. Consequently, the maximum voltage and frequency deviations, respectively, are 11 V (5% of 220) for the voltage and 0.5 Hz (1% of 50). To fine-tune the control parameters, various loading states are applied to the examined MG, as listed in Table 3. Figure 5 shows a flowchart of the GA-based optimization method. After the initialization phase, the objective function calculates initial control parameters and optimizes their performance. Finally, a new generation is produced using crossover and mutation operators after the population is sorted to find the members with the smallest voltage and frequency differences. The optimal control parameters are determined once the termination criterion has been met.

Intelligent and evolutionary algorithms are used to fine-tune control parameters in real-time to enhance the capabilities of the secondary controllers. Therefore, we develop an SCU based on ANN that interacts directly with the GA-optimization-based PI controller. To preserve the nominal set-points of frequency and voltage parameters in an online manner, initial process parameters adjusted by the GA require further additional adjustments. If the control action is ineffective, the voltage and frequency of the MG could collapse. To prevent this, we correct the control parameters using an ANN-based MG decentralized secondary control. The proposed SCUs based on ANNs modify the parameters in an online fashion, which extends the applicability of the proposed method to a more diverse set of operational conditions. The investigated system is currently collecting information on voltage and frequency fluctuations. This information is used as an ANN’s inputs and the appropriate learning rules are used to adjust the weights of the nodes controlled by the ANN. As a result, accurate set-points are generated in all of the DGs. In this manner, a risk-free control operation is accomplished, guaranteeing that the MG voltage and frequency will remain constant.

An ANN-based controller is made up of three layers: the input layer, the output layer, and the hidden layer. According to the information provided by the system expert, we decided to use five neurons for the input layer. The hidden layer contains a total of 20 neurons. In the input layer, the neurons are of a linear type, whereas the neurons in the hidden layer are nonlinear type. Because of the feature of nonlinearity, it is possible to modify the relevant weights smoothly. The number of control variables determines how many neurons are included in the output layer. As illustrated in Figure 2, the investigated MG contains five DGs. Each of these DGs has two secondary controllers, one for voltage signal and another for frequency. Every secondary controller features a proportional gain in addition to an integral gain. Because of this, the output layer has two linear neurons for every single SCU.

A neuron is the fundamental element that constitutes an ANN structure and it comprises three primary parts: the weights, which are represented by the notation $\left[w_{ij}\;w_{jk}\ldots \right]$, the bias ($\theta_{j}$), and the activation function f(net). The incoming data are denoted by the labels $x_{j}$. Equation (6) describes the relationship between these parameters [53].

(6) $$y_{j}^{p}=net_{j}^{p}=f\left(\overset{n}{\underset{i=1}{\sum }}x_{i}^{p}w_{ij}^{p}-\theta_{i}\right)\;$$

where n represents the input layer neurons,$\;w_{ij}$ denotes hidden layer weights. f(net) may be logsigmoid, sign, tansigmoid, etc. Derivatives of the activation function $\left(f^{\prime }\left(net\right)\right)$ are required for learning algorithms, such as back-propagation algorithm. Therefore, the chosen activation function must be differentiated. In addition to this, it is essential to provide an ANN-based controller with the appropriate initial conditions. The desired initial values for the voltage and frequency signals are set as the nominal values, [220 V 50 Hz].

Differential of the activation function of the hidden layer is given by

(7) $$f^{\prime }\left(net_{j}^{p}\right)=f\left(net_{j}^{p}\right)\left[1-f\left(net_{j}^{p}\right)\right]$$

The output layer nodes are calculated using

(8) $$y_{k}^{p}=net_{k}^{p}=f\left(\overset{Q}{\underset{j=1}{\sum }}y_{j}^{p}w_{jk}^{p}-\theta_{j}\right)\;\;\;\;\;\;\;\;\;\;\;\;\;\;\;\;\;k=1,2\;$$

where Q stands for the neurons in the hidden layer and $w_{jk}$ is the output layer’s weight vector.

The following equations can be used to calculate the two parameters in the PI controller:

(9) $$K_{P}^{p}=O_{1}^{p}\;$$

(10) $$K_{i}^{p}=O_{2}^{p}\;$$

When the mechanism of feed-forward is considered, the input vector is used to activate the output and hidden layers. As was previously made clear, the primary objective behind the design of the ANN structure is to minimize the already-present deviations in both frequency and voltage, as a result, to enhance the MG’s stability. The feedback procedure in this investigation makes use of the supervised learning approach to learning. The back-propagation method is used as the basis for the implementation of the learning approach. With regard to the objective of optimization, the proposed learning process makes an effort to reduce the error signal. The error function at the output of neuron *k* and *p* iteration is given by [53]:

(11) $$e_{k}^{p}=yd_{k}^{p}-y_{k}^{p}\;$$

where $y_{k}^{p}$ is the measured output variable and $yd_{k}^{p}$ is the variable denoting the desired output.

Using the error ($e_{k}^{p}$), the following updates are made to the weights.

(12) $$\;w_{jk}^{p+1}=w_{jk}^{p}+\Delta w_{jk}^{p}$$

(13) $$w_{ij}^{p+1}=w_{ij}^{p}+\Delta w_{ij}^{p}\;$$

where $\Delta w_{ij}^{p}$ and $\Delta w_{jk}^{p}$ are the weight changes caused by the system error value. The indices i, j, and k denote neurons in the input, hidden, and output layers, respectively.

(14) $$\Delta w_{jk}^{p}=\eta y_{j}^{p}\delta_{k}^{p}$$

where η, a tiny positive constant, stands for the learning rate, and at iteration p, $\delta_{k}^{p}$ represents the error gradient in the output layer’s neuron k.

When the activation function’s derivative is multiplied by the error at the neuron’s output, an error gradient is established. As a result, for output layer neuron k, we have

(15) $$\delta_{k}^{p}=\frac{\partial y_{k}^{p}}{\partial X_{k}^{p}}e_{k}^{p}$$

where $y_{k}^{p}$ represents the output of neuron k at iteration p and $X_{k}^{p}$ represents the net weighted input to neuron k at the same iteration in the process. Equation (15) can be written as for a sigmoid activation function:

(16) $$\delta_{k}^{p}=\frac{\partial \left\{\frac{1}{1+e^{-X_{k}^{p}}}\right\}}{\partial X_{k}^{p}}e_{k}^{p}=\frac{e^{-X_{k}^{p}}}{{\left\{1+e^{-X_{k}^{p}}\right\}}^{2}}e_{k}^{p}$$

As a result, we obtain:

(17) $$\delta_{k}^{p}=y_{k}^{p}\left(1-y_{k}^{p}\right)e_{k}^{p}$$

where

(18) $$y_{k}^{p}=\frac{1}{1+e^{-X_{k}^{p}}}$$

The weight updating for the hidden layer can be determined using the same formula as for the output layer:

(19) $$\Delta w_{ij}^{p}=\eta x_{i}^{p}\delta_{j}^{p}\;$$

where $\delta_{j}^{p}$ stands for the error gradient at the j neuron in the hidden layer.

(20) $$\delta_{j}^{p}=y_{j}^{p}\left\{1-y_{j}^{p}\right\}\times \overset{R}{\underset{k=1}{\sum }}\delta_{k}^{p}w_{jk}^{p}\;$$

where *R* is output layer neuron count.

(21) $$y_{j}^{p}=\frac{1}{1+e^{-\sum_{i=1}^{n}x_{i}^{p}w_{ij}^{p}-\theta_{j}}}\;$$

The learning process will keep going until the minimum error is reached.

There are two secondary outputs for each ANN- and GA-based PI secondary controller. The proposed controller’s outputs for frequency control are $\left[K_{pfANN}\;K_{ifANN}\right]$ and $\left[K_{pfs}K_{ifs}\right]$, respectively. Similarly, the output control parameters for the voltage control are $\left[K_{pvANN}\;K_{ivANN}\right]$ and $\left[K_{pvs}K_{ivs}\right]$.

(22) $$\Delta V_{sec}=K_{pvANN}K_{pvs}\times E_{v}+K_{ivANN}K_{ivs}\times \int^{\;}E_{v}\;$$

(23) $$\Delta f_{sec}=K_{pfANN}K_{pfs}\times E_{f}+K_{ifANN}K_{ifs}\times \int^{\;}E_{f}\;$$

where $E_{v}$ and $E_{f}$ are the error signals of the voltage and frequency, respectively. The error signal represents the difference between the reference value and the measured one.

## 5. Decentralized Secondary Control Formulation

**Figure 6:**
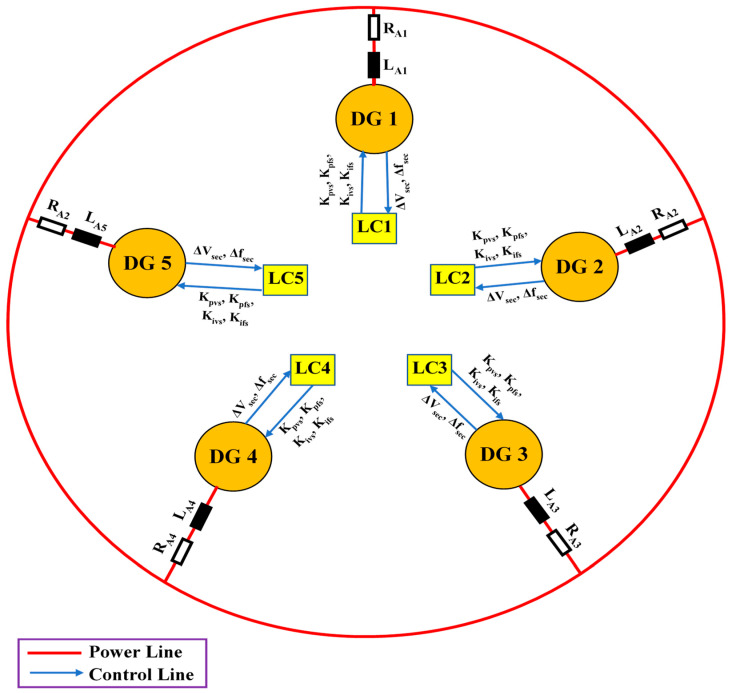
Intelligent decentralized secondary control structure.

In the proposed decentralized secondary control, every DG unit has an SCU to remedy voltage and frequency deviations and to ensure that the adopted parallel DGs are properly sharing active and reactive power. Figure 6 illustrates the proposed system’s intelligent decentralized secondary control structure.

As depicted in Figure 3, the outputs of the power controller are given in Equations (24) and (25):

(24) $$\omega =-mP+\omega_{ref}+\Delta f_{sec}$$

(25) $$V_{dref}=-nQ+V_{ref}+\Delta V_{sec}\;,\;V_{dref}=0$$

where ω is the frequency in (rad/sec.), P and Q are the measured signals of both active and reactive power of the related DG, $\omega_{ref}$ and $V_{ref}$ are the reference values of the MG frequency and voltage, $\Delta f_{sec}$ and $\Delta V_{sec}$ are the frequency and voltage deviations (outputs of the SCUs), and $V_{dref}$ and $V_{qref}$ are the dq-frame reference voltages for the voltage controllers.

The secondary outputs are introduced to the conventional droop equations to be able to suppress any deviations in voltage and frequency. To ensure the DGs share the load power equally, virtual impedance loops were embedded in the primary control using Equations (26) and (27):

(26) $$V_{dver}=-\omega_{ref}L_{v}I_{q}+R_{v}I_{d}$$

(27) $$V_{qver}=-\omega_{ref}L_{v}I_{d}+R_{v}I_{q}$$

where $R_{v}$ and $L_{v}$ are the virtual resistance and inductance, $I_{d}$ and $I_{q}$ are the dq-frame inverter currents, and $V_{dver}$ and $V_{qver}$ are the drop voltages compensation due to mismatched line impedances.

The outer voltage controller is used for supplying the reference dq-frame currents ($I_{dref}$ and $I_{qref}$) to the inner current controller using Equations (28) and (29)

(28) $$I_{dref}=\left\{\left(V_{dref}-V_{dinv}-V_{dver}\right)K_{pv}+\int^{\;}\left(V_{dref}-V_{dinv}-V_{dver}\right)K_{iv}\right\}-\omega_{ref}C_{f}V_{qinv}+I_{d\;}$$

(29) $$I_{qref}=\left\{\left(V_{qref}-V_{qinv}-V_{qver}\right)K_{pv}+\int^{\;}\left(V_{qref}-V_{qinv}-V_{qver}\right)K_{iv}\right\}-\omega_{ref}C_{f}V_{dinv}+I_{q}$$

where $V_{dinv}$ and $V_{qinv}$ are the dq-frame inverter voltages, $K_{pv}$ and $K_{iv}$ are the control parameters of the voltage PI controller, and $\omega_{ref}C_{f}V_{dinv}$ and $\omega_{ref}C_{f}V_{qinv}$ are the cross-decoupled quantities that are used to control the voltage independently along the dq axis.$\;C_{f}$ is the output filter capacitor.

The inner current controller produces the reference inverter voltage in dq-frame ($V_{dref,inv}$ and $V_{qref,inv}$) using Equations (30) and (31):

(30) $$V_{dref,inv}=\left\{\left(I_{dref}-I_{d}\right)K_{pi}+\int^{\;}\left(I_{dref}-I_{d}\right)K_{ii}\right\}-\omega_{ref}L_{f}I_{q}$$

(31) $$V_{qref,inv}=\left\{\left(I_{qref}-I_{q}\right)K_{pi}+\int^{\;}\left(I_{qref}-I_{q}\right)K_{ii}\right\}-\omega_{ref}L_{f}I_{d}\;$$

where $K_{pv}$ and $K_{iv}$ are the control parameters of the current PI controller, while $\omega_{ref}L_{f}I_{q}$ and $\omega_{ref}L_{f}I_{d}$ are the cross-decoupled quantities that are used to control the current independently along the dq axis. Lastly, $L_{f}$ is the output filter inductor.

The voltage reference values of the inverter in dq0 frame are then transformed into abc frame using the inverse of Park transformation as follows:

(32) $$\left[\begin{array}{c} V_{a}\\ V_{b}\\ V_{c} \end{array}\right]=\left[\begin{array}{ccc} \cos\left(\omega t\right) & -\sin\left(\omega t\right) & 1\\ \cos\left(\omega t-\frac{2\pi }{3}\right) & -\sin\left(\omega t-\frac{2\pi }{3}\right) & 1\\ \cos\left(\omega t+\frac{2\pi }{3}\right) & -sin\left(\omega t-\frac{2\pi }{3}\right) & 1 \end{array}\right]\left[\begin{array}{c} V_{dref,inv}\\ V_{qref,inv}\\ 0 \end{array}\right]$$

where frequency in radian (ω) is given in Equation (24).

Then, these three phase signals are passed through the Pulse Width Modulation (PWM) generator to trigger the power electronic switches of the related VSI.

## 6. Simulation Results and Discussion

The proposed islanded MG illustrated in Figure 2 is performed under load changes to evaluate the performance of the proposed control structure. After applying load changes to the MG, the system response, consisting of frequency and voltage profiles, is determined in three scenarios. The first scenario is to test the system without adopting SCUs, the second one is by using GA-based SCUs, and the third scenario is by using proposed GA+ANN-based SCUs.

**Figure 7:**
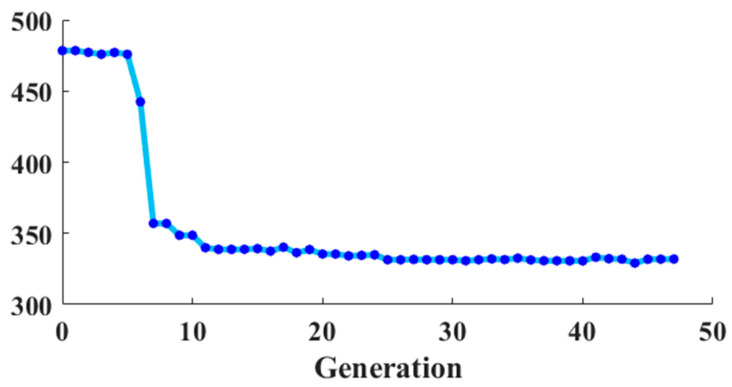
The GA-based convergence curve for the system under investigation.

**Figure 8:**
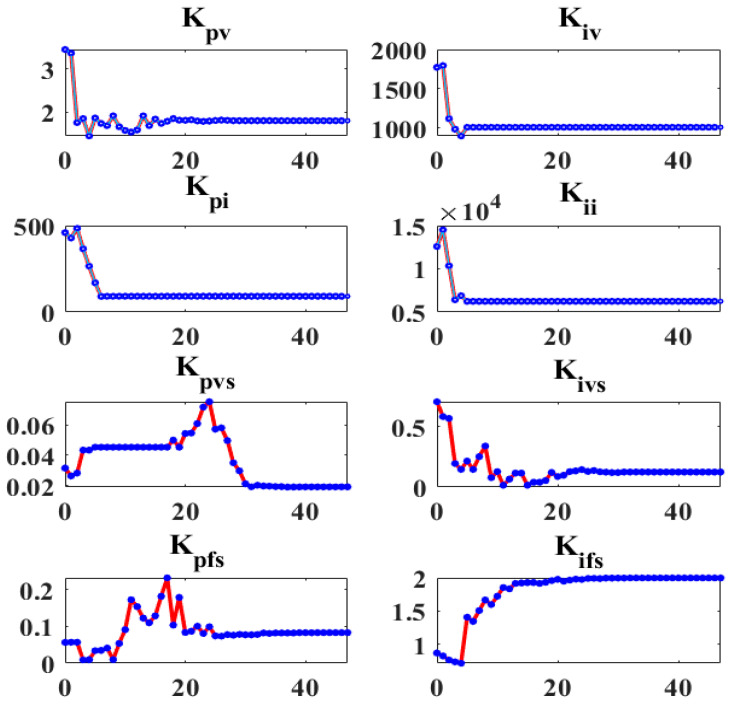
The GA-based optimum values of control parameters.

Figure 7 illustrates the convergence curve of the GA for the investigated MG system. Evidently, the problem is approaching its optimal solution. Consequently, the optimum control parameters shown in Figure 8 are attained, where [K_pi_ K_ii_] are the proportional and integral control parameters of the current controller-based primary control, [K_pv_ K_iv_] are the voltage controller parameter-based primary control, [K_pvs_ K_ivs_] are the secondary voltage controller, and [K_pfs_ K_ifs_] are the secondary frequency controller parameters. A quick time of convergence is not required and waiting longer may result in better control parameters, as this is the first offline phase of the proposed method. The convergence and optimal parameter values are obtained by making the simulation time 5.5 s and setting the GA parameters as follows: number of population = 10, number of generations = 50, the lower limits are all “0”, upper limit = [10 4000 1000 40,000 0.1 2 0.5 2]. The required elapsed time is 31,998 s for the GA to obtain the control parameters’ optimal values using the mentioned specifications.

**Figure 9:**
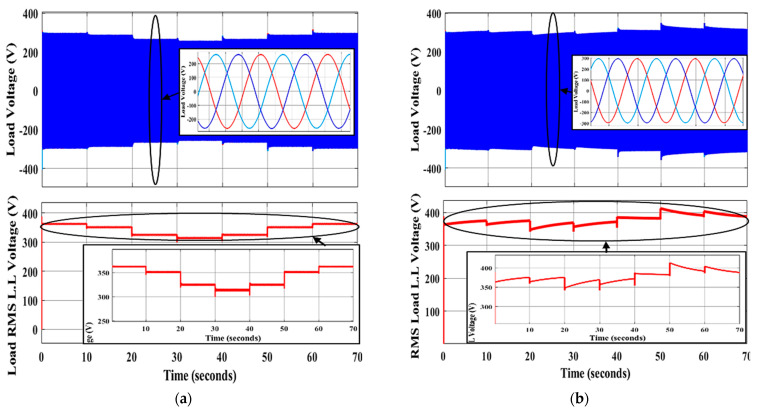
The system voltage using primary control in (**a**) and with applying GA-based SCUs in (**b**).

**Figure 10:**
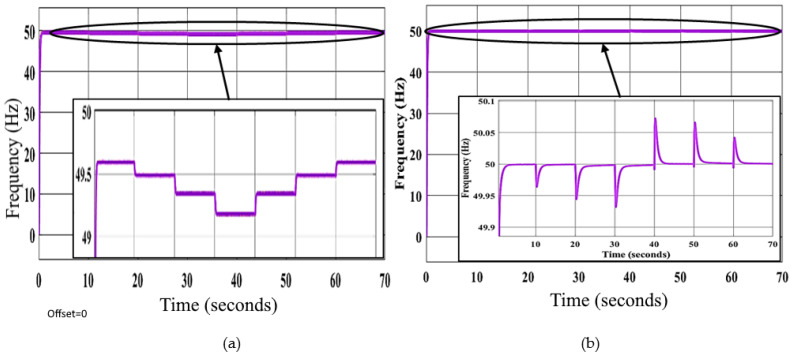
The system frequency response using primary control in (**a**) and with applying GA-based SCUs in (**b**).

The voltage and frequency responses of the system under droop-control-based primary control and GA-based secondary control are shown in Figure 9a and Figure 10a, respectively. It is clear that the system is not robust in the first case (primary control only) when the load varies, as shown in Table 3. Voltage and frequency deviations induced by a changed load lead these parameters to deviate from their nominal values. As shown in Figure 9b, the system voltage is not robust. The controller cannot return to the reference voltage properly during load changes in the second scenario (using SCUs based on GA-based offline tuning parameters). However, the system frequency is acceptable and is attained to the nominal value, as shown in Figure 10b, due to it being a global parameter.

**Figure 11:**
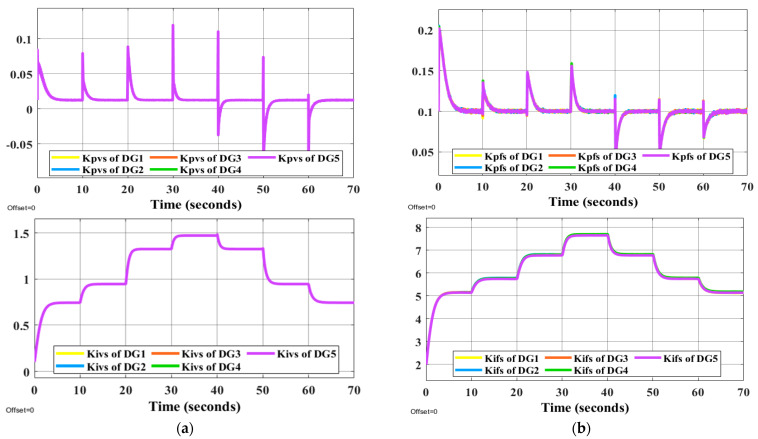
Updating the (**a**) voltage and (**b**) frequency parameters of the SCUs in DGs after the variations in loading conditions using ANN.

In the third scenario, the ANNs with GA-optimized PI controllers based on the proposed method alter the SCU parameters in all DGs to respond to the operating point changes to stabilize the frequency and voltage signals at their set-points. These parameter-updated curves in all SCUs of the control system are shown in Figure 11. These data demonstrate that ANN-based SCUs modify all DGs’ control parameters to achieve the lowest possible voltage and frequency profile fluctuations.

**Figure 12:**
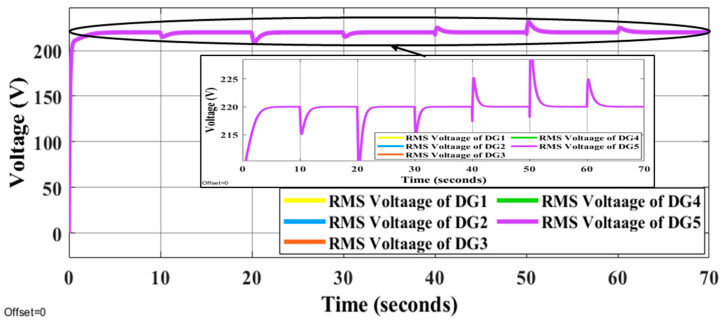
Voltage responses of all DGs using proposed online method under the step changes in loads.

**Figure 13:**
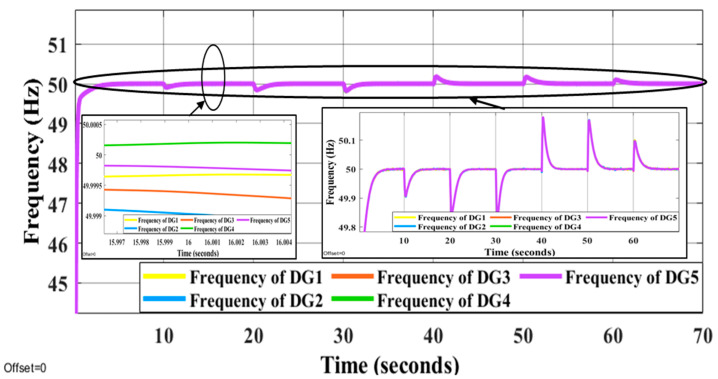
Frequency patterns of all DGs under using proposed online method under the step changes in loads.

**Figure 14:**
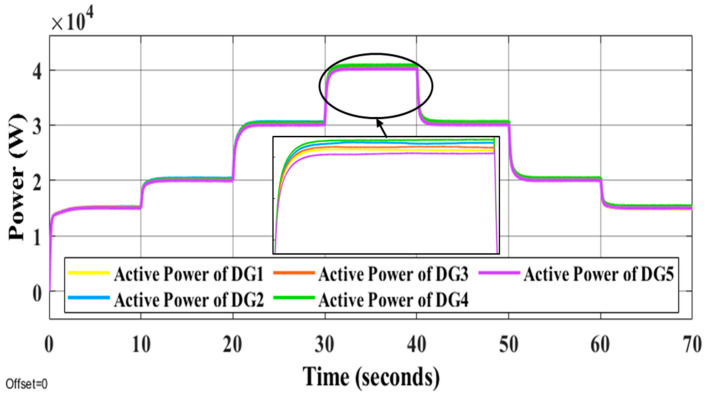
Active power of all DGs under the changing of load conditions.

**Figure 15:**
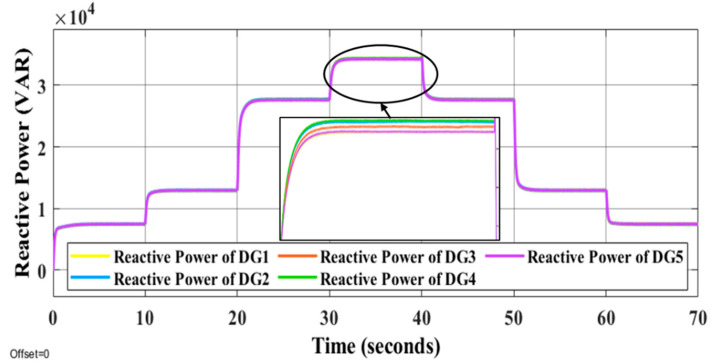
Reactive power of all DGs under the changing of load conditions.

It is evident that the proposed methods have the desired performance under load changes in terms of voltage and frequency deviation minimization (steady-state errors). Figure 12 and Figure 13 display the voltage and frequency of every DG, respectively. It can be seen that the proposed control method is robust against load changes. The voltage and frequency are settled at the nominal values (50 Hz and 220 V RMS phase voltage). Figure 14 and Figure 15 show the active and reactive power-sharing outcomes using the proposed techniques. By adopting a virtual-impedance-based proposed control structure, the five DGs are balanced for both transient and steady-state load sharing and all VSIs follow one another. Therefore, the load power at any load step change is divided equally by five according to the number of adopted DGs. Table 4 compares the conventional droop control with the alternatives proposed.

**Figure 16:**
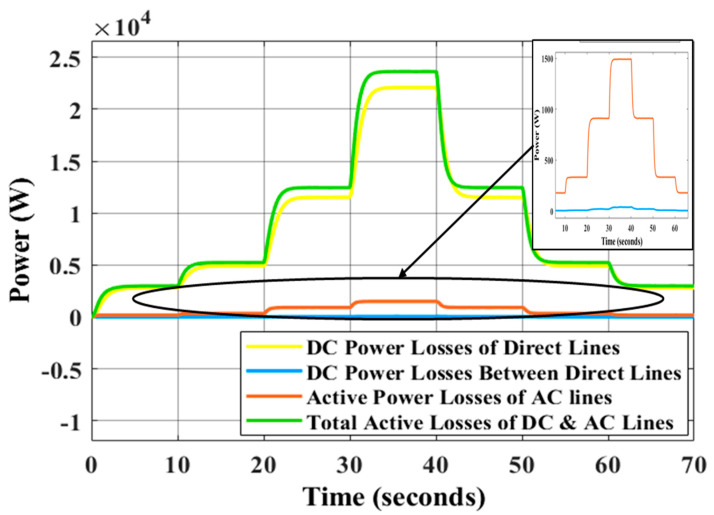
Active power losses of MG under the changing of load conditions using high resistances for DC lines.

**Figure 17:**
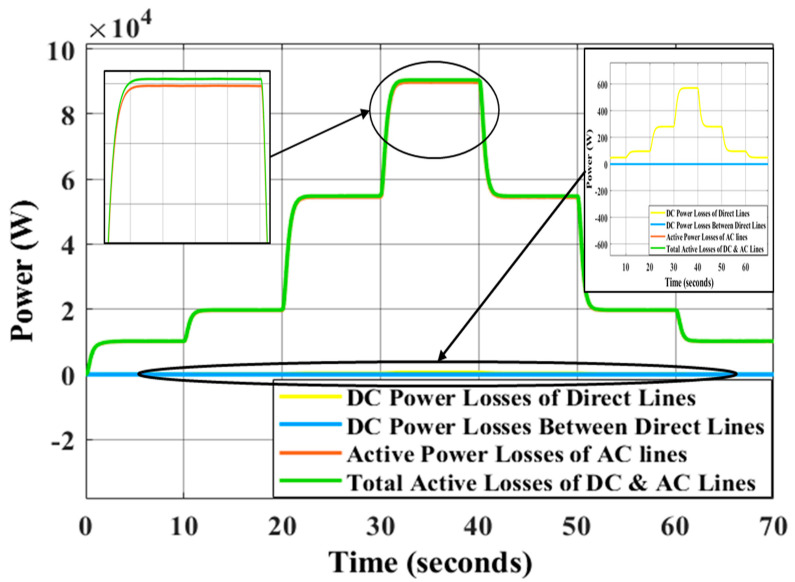
Active power losses of MG under the changing of load conditions using high resistances for AC lines.

**Figure 18:**
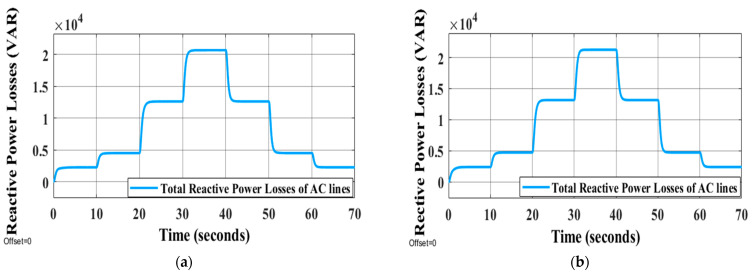
Reactive power losses of AC transmission lines under the changing of load conditions using (**a**) high resistances for DC lines and (**b**) using high resistances for AC lines.

The traditional droop approach shows the inability to restore frequency and voltage at a steady state and improper sharing of active and reactive power is seen in Table 4 for the traditional droop approach. Adopting ANN and GA allows the GA tuning-based control parameter to be adjusted in real time continuously, whereas proposing secondary controllers based on GA for offline tuning parameters does not correctly restore the system’s voltage to nominal value because the control parameters are not adjusted simultaneously. As a result, the active and reactive powers are distributed fairly and the voltage and frequency may return to their normal levels. The difference between the power of DGs and load power is identified as power losses in the system caused by the impedances of the transmission lines. Figure 16 illustrates the active power losses of the MG’s lines using LVDC transmission with the impedances of lines shown in Table 1. As can be seen in Table 1, the impedance of the AC transmission lines is significantly lower than that of the DC transmission lines. AC lines are adopted for short distances, while DC lines are used for long ones because the DC line impedance is primarily resistive in low-voltage wiring [54,55]. In Figure 17, the active power losses of the MG transmission lines are shown and this figure is not deduced using the line impedances in Table 1; rather, the resistance values of DC lines and AC lines are exchanged (the high resistance of DC lines is placed instead of the resistance of AC lines and vice versa). The purpose of resistance exchange is to observe and compare the active power losses obtained by long-distance DC transmission (Figure 16) with transmission via AC lines (Figure 17). It is evident from Figure 16 that the total active power losses in the DC lines are shown in yellow and blue curves. The blue curve is related to the losses of the lines between the main or direct DC transmission lines and it is close to zero watt as a result of the fact that the DC drop voltage across each resistance ($R_{d1,2},R_{d2,3},\;R_{d3,4},R_{d4,5},\;and\;R_{d5,1}$) in these lines is close to zero volt. Under the step changes in the loads, the main or direct DC lines are represented by the yellow curve in the figure. This losses are equal to approximately 25% of the power losses when transmitting electricity by the AC lines, as shown in Figure 17 (red curve). In Figure 18, the AC transmission also suffers reactive power losses. The reactive power losses in Figure 18a,b have low differences because the line inductances are the same with long-distance DC transmission or AC transmission, respectively.

## 7. Conclusions

In this paper, a robust decentralized MG control was proposed. This MG system uses LVDCT to decrease power losses and remove reactive power issues, while short-distance AC transmission lines provide MG AC loads. This research offered new ANNs and GA-based SCUs as well as a changed principal control structure, employing a virtual impedance approach with GA-optimized PI controller parameters. When load changes occur, typical PI controllers cannot operate safely. Increasing load variations may induce MG instability. Using offline methods, such as GAs or ANNs, to individually adjust frequency or voltage does not achieve instantaneous tuning. The proposed method adjusts frequency and voltage simultaneously, improving system operating indices. GA first guided the parameter adjustments. This strategy improved the MG’s performance but caused steady-state errors. The ANN method to fine-tune control parameters online solved this issue. GA-based decentralized control improved ANNs’ learning and extensibility. Our controller reduces frequency and voltage profile variations, regardless of the operating point. Thus, steady-state errors were reduced and the proposed system ran normally despite disruptions. The proposed method can manage a larger range of operating points and enhance power equality, making it appropriate for MGs.

Possible future work on this subject might entail applying the proposed secondary control with a consensus method to govern and distribute frequency, active power, voltage, and reactive power of each DG with its neighbors. The future secondary controller will suit any MG architecture, including mesh and ring configurations. Eventually, practical applications and deployment in specific environments, such as ambient intelligence systems or smart environments, might be considered [56].

## Figures and Tables

**Table 1:** The MG line impedances.

AC Line Impedance	Value (Ω)	DC Line Impedance	Value (Ω)	DC Line Length (km)	Impedance between DC Lines	Value (Ω)
*R_A_*_1_ + jX_A1_	0.01273 + j0.219	*R_d_*_1_	1	100	*R_d_*_1,2_	0.0127
*R_A_*_2_ + jX_A2_	0.0159125 + j0.2748	*R_d_*_2_	0.95	92	*R_d_*_2,3_	0.0317
*R_A_*_3_ + jX_A3_	0.016549 + j0.2858	*R_d_*_3_	0.76	80	*R_d_*_3,4_	0.0317
*R_A_*_4_ + jX_A4_	0.019095 + j0.3298	*R_d_*_4_	1.27	125	*R_d_*_4,5_	0.0127
*R_A_*_5_ + jX_A5_	0.014003 + j0.2419	*R_d_*_5_	1.14	115	*R_d_*_5,1_	0.0381

**Table 2:** The adopted droop coefficients and power filter parameters.

VSI	Parameter Name	Value
VSI 1 and VSI 2	Frequency Droop Coefficient	9.5 × 10^−5^
	Voltage Droop Coefficient	1.3 × 10^−3^
	Power Filter Resistance	0.1 Ω
	Power Filter Inductance	1.35 mH
	Power Filter Capacitance	500 µf
VSI 3, VSI 4 and VSI 5	Frequency Droop Coefficient	12.5 × 10^−5^
	Voltage Droop Coefficient	1.5 × 10^−3^
	Power Filter Resistance	0.1 Ω
	Power Filter Inductance	1.35 mH
	Power Filter Capacitance	500 µf

**Table 3:** Time-value-based active and reactive load power.

Time Duration (s)	Activating Load	Load Value (W + jVAR)
0–10 s	Load 1	75,000 + j35,000
10–20 s	Load 1 + Load 2	100,000 + j60,000
20–30 s	Load 1 + Load 2 + Load 3	150,000 + j125,000
30–40 s	Load 1 + Load 2 + Load 3 + Load4	200,000 + j150,000
40–50 s	Load 1 + Load 2 + Load 3	150,000 + j125,000
50–60 s	Load 1 + Load 2	100,000 + j60,000
60–70 s	Load 1	75,000 + j35,000

**Table 4:** Traditional droop control vs. proposed alternatives.

	Frequency Restoration	Voltage Restoration	Active Power Sharing	Reactive Power Sharing
Droop Control (without Secondary control)	✗	✗	✗	✗
Offline Parameters Tuning-Based Secondary Control	✓	✓ > ✗	✓	✓
Proposed Online Parameters Tuning-Based Secondary Control	✓	✓	✓	✓

## Data Availability

Not applicable.
